# Moderate and high amounts of tamoxifen in *αMHC-MerCreMer* mice induce a DNA damage response, leading to heart failure and death

**DOI:** 10.1242/dmm.010447

**Published:** 2013-08-07

**Authors:** Kevin Bersell, Sangita Choudhury, Mariya Mollova, Brian D. Polizzotti, Balakrishnan Ganapathy, Stuart Walsh, Brian Wadugu, Shima Arab, Bernhard Kühn

**Affiliations:** 1 Department of Cardiology, Boston Children’s Hospital, MA 02115, USA; 2 Department of Pediatrics, Harvard Medical School, Boston, MA 02115, USA; 3 Harvard Stem Cell Institute, Cambridge, MA 02138, USA

## Abstract

Numerous mouse models have utilized Cre-*loxP* technology to modify gene expression. Adverse effects of Cre recombinase activity have been reported, including in the heart. However, the mechanisms associated with cardiac Cre toxicity are largely unknown. Here, we show that expression of Cre in cardiomyocytes induces a DNA damage response, resulting in cardiomyocyte apoptosis, cardiac fibrosis and cardiac dysfunction. In an effort to increase the recombination efficiency of a widely used tamoxifen-sensitive *Cre* transgene under control of the α-myosin-heavy-chain promoter (*αMHC-MerCreMer*), we observed myocardial dysfunction and decreased survival, which were dependent on the dose of tamoxifen injected. After excluding a Cre-independent contribution by tamoxifen, we found that Cre induced myocardial fibrosis, activation of pro-fibrotic genes and cardiomyocyte apoptosis. Examination of the molecular mechanisms showed activation of DNA damage response signaling and p53 stabilization in the absence of *loxP* sites, suggesting that Cre induced illegitimate DNA breaks. Cardiomyocyte apoptosis was also induced by expressing Cre using adenoviral transduction, indicating that the effect was not dependent on genomic integration of the transgene. Cre-mediated homologous recombination at *loxP* sites was dose-dependent and had a ceiling effect at ∼80% of cardiomyocytes showing recombination. By titrating the amount of tamoxifen to maximize recombination while minimizing animal lethality, we determined that 30 μg tamoxifen/g body weight/day injected on three consecutive days is the optimal condition for the *αMHC-MerCreMer* system to induce recombination in the *Rosa26-lacZ* strain. Our results further highlight the importance of experimental design, including the use of appropriate genetic controls for Cre expression.

## INTRODUCTION

The use of Cre recombinase, encoded by the bacteriophage P1, to catalyze recombination between engineered *loxP* sites is an important tool for regulating gene expression (Nagy, 2000). Side effects of Cre expression have been observed in a variety of tissues (Schmidt-Supprian and Rajewsky, 2007). Cre can induce illegitimate chromosome rearrangements, micronuclei formation and other forms of DNA alterations independent of *loxP* sites (Thyagarajan et al., 2000; Semprini et al., 2007), leading to reduced proliferation and increased apoptosis in a variety of cells, including fibroblasts, pneumocytes and cells of the hematopoietic lineage (Loonstra et al., 2001; Jeannotte et al., 2011).

We used a transgenic mouse strain that allows for the temporal control of the nuclear translocation of Cre in cardiomyocytes (Sohal et al., 2001), which is achieved with a fusion construct of Cre recombinase and the modified estrogen receptor (Mer) (Zhang et al., 1996), expressed under control of the α-myosin heavy chain promoter (*αMHC*) (Sohal et al., 2001). Injecting the estrogen receptor ligand tamoxifen induces nuclear translocation of Cre, which results in site-specific recombination of *loxP* sequences. The reported recombination efficiency for this *αMHC-MerCreMer* strain is ∼80% (Sohal et al., 2001; Hsieh et al., 2007). To increase this recombination efficiency, we tested a range of tamoxifen doses, which we selected based on published protocols, namely 20 μg/g body weight (Sohal et al., 2001) and 80 μg/g body weight (Li et al., 2005). We observed toxic effects at the tissue and cellular level, which have not been previously reported. Our more detailed analyses showed that some of these changes were also present with standard doses of tamoxifen.

Cardiac toxicity due to Cre activity has been reported (Buerger et al., 2006; Hall et al., 2011; Koitabashi et al., 2009; Hougen et al., 2010) and reviewed (Molkentin and Robbins, 2009), but the underlying mechanisms remain largely unknown. The initial report of cardiac toxicity used a constitutively active *Cre* transgene under control of the *αMHC* promoter (Buerger et al., 2006). A subsequent study using the same *αMHC-MerCreMer* transgene that we applied in this study reported a transient reduction of systolic cardiac function (Koitabashi et al., 2009), which could be circumvented with an alternative protocol using raloxifen. Gene dysregulation upon activation of the *αMHC-MerCreMer* system was found in several studies (Koitabashi et al., 2009; Hall et al., 2011; Hougen et al., 2010), but the underlying cellular and molecular mechanisms in cardiomyocytes remain poorly defined. More specifically, a possible effect on apoptosis in the *MerCreMer* system has been described in other tissues (Naiche and Papaioannou, 2007; Forni et al., 2006) but has not been investigated in the heart. The present study provides insight into the potential cellular and tissue mechanisms of cardiac Cre toxicity, and offers a way to minimize these detrimental effects while achieving maximal Cre recombination efficiency.

**TRANSLATIONAL IMPACT**

**Clinical issue**

The prokaryotic recombinase Cre is a crucial tool in animal model research. This genetic system allows for temporal modification of gene expression, and has enabled researchers to build an expansive knowledge of the roles of many mammalian genes in biology and disease. Recently, there has been increasing interest in genome editing technologies. Genes delivered as therapeutics can be flanked by *loxP* sites in order to be excised by Cre after the desired effect has taken place, to provide more targeted, controlled therapies with minimal adverse effects. However, previous studies have reported negative effects associated with Cre-*loxP* technology, so it is important to fully understand the potential toxicities before this system can be launched in the clinical arena.

**Results**

Cre-recombinase-associated toxicities have been reported in the heart in animal models of cardiac disease. To investigate the extent of these negative effects, the authors utilized a transgenic mouse strain in which Cre recombinase was expressed specifically in cardiomyocytes. They report that Cre activity leads to cardiac toxicity and death in a dose-dependent manner. Tamoxifen alone had no effect on cardiac structure or function, regardless of the dose used. In the presence of high levels of Cre activity, approximately 50% of the animals died within 2 weeks of beginning the protocol. Echocardiography revealed decreased cardiac function in the absence of organ-level structural changes. Histological evaluation at the tissue level showed increased cardiac fibrosis at high doses of tamoxifen, correlating with high levels of Cre activity. Cardiomyocyte cell death was associated with Cre recombinase activity, as evidenced by stabilization of p53 and activation of a DNA damage response. Finally, the authors show that the cellular mechanism of cardiomyocyte apoptosis precedes fibrosis formation.

**Implications and future directions**

This report highlights the need for careful titration of experimental Cre recombinase use in designing each experiment. There is a unique response for each tissue type at a variety of Cre levels. The inclusion of Cre recombinase controls in all animal models should now be established as a must when utilizing this tool for genetic manipulations. The study also provides evidence for activation of the DNA damage response and apoptosis as early events that are associated with Cre recombinase activity in cardiomyocytes, which could indicate that these processes contribute to the onset of myocardial fibrosis.

## RESULTS

### Cre causes dose-dependent lethal heart failure

**Fig. 1.**
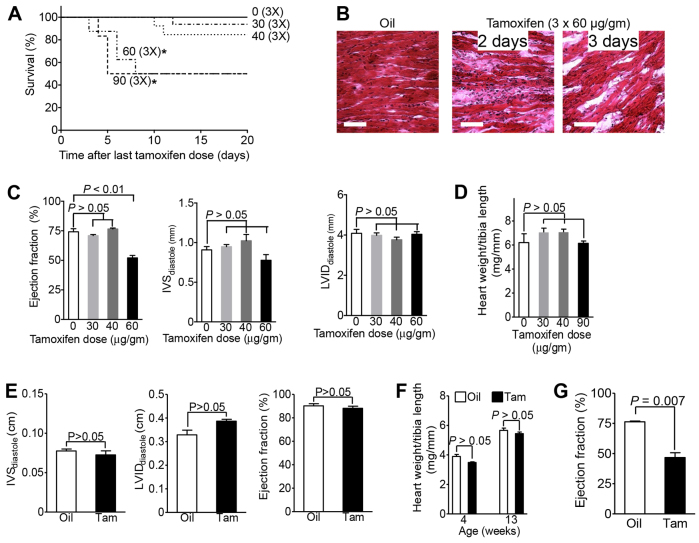
**Tamoxifen injections cause dose-dependent lethal heart failure in *αMHC-MerCreMer* mice.** Tamoxifen was given in three doses as indicated in μg/g body weight. (A) Tamoxifen induces dose-dependent mortality within 1 week of injection. Mortality at doses of 60 and 90 μg/g body weight was statistically significantly different from no tamoxifen (*P*<0.02 by logrank test, asterisks). (B) Myocardial disorganization and accumulation of extracellular material in moribund mice that were euthanized. Scale bars: 100 μm. (C) Echocardiography performed 1 week after completion of tamoxifen treatment shows diminished myocardial function at 3×60 μg/g body weight. (D) Cre induction does not result in cardiac hypertrophy. (E,F) C57/BL6 mice received five doses of tamoxifen of 90 μg/g body weight. Cardiac structure or function, determined by echocardiography 2 weeks after injection of tamoxifen (E), and heart weight (F) were unchanged, indicating that tamoxifen alone was not toxic. (G) Echocardiography performed 4 weeks after completion of tamoxifen administration (3×60 μg/g body weight) shows diminished myocardial function. *n*≥4 animals per group (A,C–G), *n*=2 (G, oil); statistical significance determined with ANOVA (C,D,F) and *t*-test (E,G).

We injected tamoxifen intraperitoneally on three consecutive days at doses between 0 and 90 μg/g body weight/day in 6-week-old mice. We were surprised to observe death in the *αMHC-MerCreMer* strain at tamoxifen doses of 60 and 90 μg/g body weight. To determine whether this mortality was associated with the given dose of tamoxifen, we analyzed Kaplan-Meier curves (Fig. 1A). No death occurred after injecting one single dose of 1 or 5 μg tamoxifen/g body weight (Fig. 1A). Injecting 3×30 μg tamoxifen/g body weight caused 10% mortality [*P*>0.05 compared with oil injection (control)] and 3×40 μg tamoxifen/g body weight was associated with 18% mortality (*P*>0.05 compared with oil; Fig. 1A). Injecting 3×60 and 3×90 μg tamoxifen/g body weight caused 50% mortality within 7.5 days (*P*<0.02 and *P*<0.05, respectively, compared with oil; Fig. 1A). At 1–2 days prior to death, some of these mice showed severely decreased activity and hypothermia. These severely ill-appearing mice were euthanized 2–6 days after tamoxifen administration. Their hearts appeared soft and dilated, and the myocardium was inflamed, swollen and disorganized (Fig. 1B). In summary, the incidence of early mortality when inducing Cre activity in cardiomyocytes was dependent on the tamoxifen dose.

Because of the quick onset of this lethal phenotype and the associated signs and symptoms, we hypothesized that the underlying mechanisms might involve diminished cardiac function. We assessed myocardial function by echocardiography in *αMHC-MerCreMer* mice in the absence of tamoxifen, observing normal function (data not shown), which is consistent with published data (Sohal et al., 2001). However, at a tamoxifen dose of 60 μg/g body weight for three consecutive days, fractional shortening (FS) was significantly decreased, by 22.1% (compared with 0 μg/g, *P*<0.01), in the surviving mice (Fig. 1C). At the lower doses, the mice had normal cardiac function (Fig. 1C). We did not observe cardiac hypertrophy, determined by echocardiographic measurement of the interventricular septal (IVS) thickness, or ventricular dilation, determined by echocardiographic measurement of the left ventricular (LV) internal dimension (LVID) (Fig. 1C). The lack of cardiac hypertrophy was further supported by a normal heart weight:tibia length ratio (Fig. 1D). In summary, nuclear Cre activity led to diminished cardiac function in a dose-dependent fashion without cardiac hypertrophy or dilation, although transient cardiac hypertrophy was documented in one other study (Hall et al., 2011).

It is possible that tamoxifen might be directly toxic. We therefore determined whether our tamoxifen injection protocol resulted in cardiac toxicity in wild-type C57/BL6 mice by injecting the highest dose of tamoxifen, 90 μg/g body weight, on five consecutive days. Echocardiography showed that the IVS thickness and the LV dimension in diastole were not different between tamoxifen-injected and oil-injected (control) mice (Fig. 1E). The ejection fraction also was unchanged, indicating normal myocardial function (Fig. 1E). In addition, the heart weight:tibia length ratios were not different between control and tamoxifen-injected mice, indicating a lack of organ hypertrophy (Fig. 1F). In summary, injecting a large amount of tamoxifen in adult C57/BL6 mice did not reproduce the observed effects.

One prior study reported that tamoxifen induction of the *αMHC-MerCreMer* strain induces transient myocardial dysfunction, which was resolved after 2 weeks (Koitabashi et al., 2009). To determine whether the myocardial dysfunction that we observed in mice surviving after injection of 3×60 μg tamoxifen/g body weight was transient, we performed echocardiography at 4 weeks (supplementary material Movies 1, 2). This showed a significant reduction of the ejection fraction by 30% (*P*=0.007; Fig. 1G).

### High levels of Cre induction lead to myocardial fibrosis

**Fig. 2.**
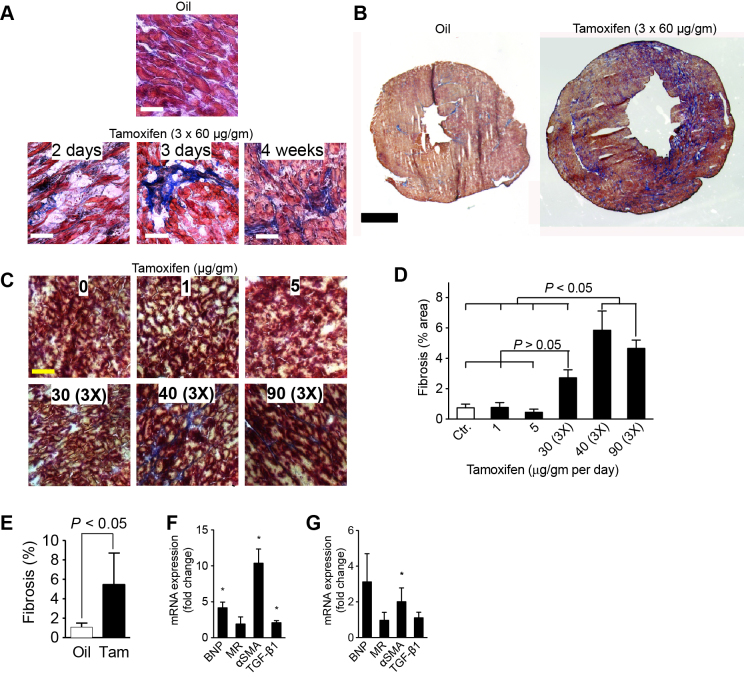
**Tamoxifen injections induce fibrosis in *αMHC-MerCreMer* mice.** Mice received tamoxifen at the indicated doses in μg/g body weight and frequencies. Fibrosis was visualized with AFOG staining. (A) Earliest evidence of fibrosis, 2 and 3 days after tamoxifen injections (3×60 μg/g body weight). (B) Representative cross-sections showing regional fibrosis 4 weeks after injection of 3×60 μg/g body weight tamoxifen. (C) Examples of fibrosis development 2 weeks after tamoxifen administration at the indicated doses and frequencies. (D) Quantification of multiple mice and sections shows that Cre leads to fibrosis after tamoxifen doses above 30 μg/g body weight. (E) Increased myocardial fibrosis 4 weeks after administration of 3×60 μg tamoxifen/g body weight. Scale bars: 100 μm (A,C) and 1 mm (B). *n*≥4 animals per group (C–E). (F,G) Quantitative RT-PCR reveals an increase of pro- and anti-fibrotic markers, normalized to GAPDH expression, in tamoxifen- compared with oil-injected animals 2 weeks after tamoxifen injection of 3×30 μg/g body weight (F, *n*=4) and 4 weeks after injection of 3×60 μg/g body weight (G, *n*=8): brain natriuretic peptide (BNP), mineralocorticoid receptor (MR), α-smooth muscle actin (α-SMA) and transforming growth factor-β1 (TGF-β1). Statistical significance tested by ANOVA (D) and *t*-test (E–G). *P<0.05.

Because cardiac function in surviving mice was diminished without noticeable hypertrophy or dilation, we hypothesized that the myocardium became less compliant, possibly by fibrosis. We noticed foci of myocardial fibrosis in moribund mice that were euthanized 2 days after completion of 3×60 μg tamoxifen/g body weight (Fig. 2A). However, the global extent of fibrosis was very mild at this point. At 4 weeks after administration of 3×60 μg tamoxifen/g body weight, myocardial fibrosis was present in an inhomogenous pattern (Fig. 2B). We then examined myocardial fibrosis 2 weeks after administration of different doses and frequencies of tamoxifen, when cardiac function was known to be depressed (Fig. 2C). Oil-injected *αMHC-MerCreMer*^+/+^ mice (control) had myocardial fibrosis in 0.7±0.2% of the left ventricle (Fig. 2D). Mice injected with single doses of 1 or 5 μg/g body weight had no change in fibrotic area compared with controls, and three injections of 30 μg tamoxifen/g body weight resulted in 2.7±0.5% of the myocardial area being fibrotic (*P*>0.05; Fig. 2D). However, surviving animals that had received three doses of 40 and 90 μg tamoxifen/g body weight had ∼5% fibrosis, a statistically significant increase over doses of ≤30 μg/g body weight (Fig. 2D). To determine whether this fibrosis was sustained, we examined a group of mice at 4 weeks after injecting 3×60 μg tamoxifen/g body weight. Compared with oil-injected littermate controls, there was significantly increased fibrosis (Fig. 2E). In conclusion, Cre activity results in cardiac fibrosis after tamoxifen treatment at doses >30 μg/g body weight.

Myocardial fibrosis is associated with activation of a transcriptional profile, including brain natriuretic peptide (*BNP*), mineralocorticoid receptor (*MR*), α-smooth muscle actin (*α-SMA*) and transforming growth factor-β1 (*TGF-β1*). We tested whether these genes might be activated in mice that showed Cre-dependent fibrosis. The relative expression of *BNP*, *α-SMA* and *TGF-β1* was elevated 2 weeks after injection of 3×30 μg tamoxifen/g body weight (Fig. 2F). At 4 weeks after injection of 3×60 μg/g body weight, the transcription of *α-SMA* was elevated (Fig. 2G). In summary, Cre-dependent myocardial fibrosis is accompanied by changes in transcription of pro- and antifibrotic genes.

### High levels of Cre activity lead to cardiomyocyte apoptosis

**Fig. 3.**
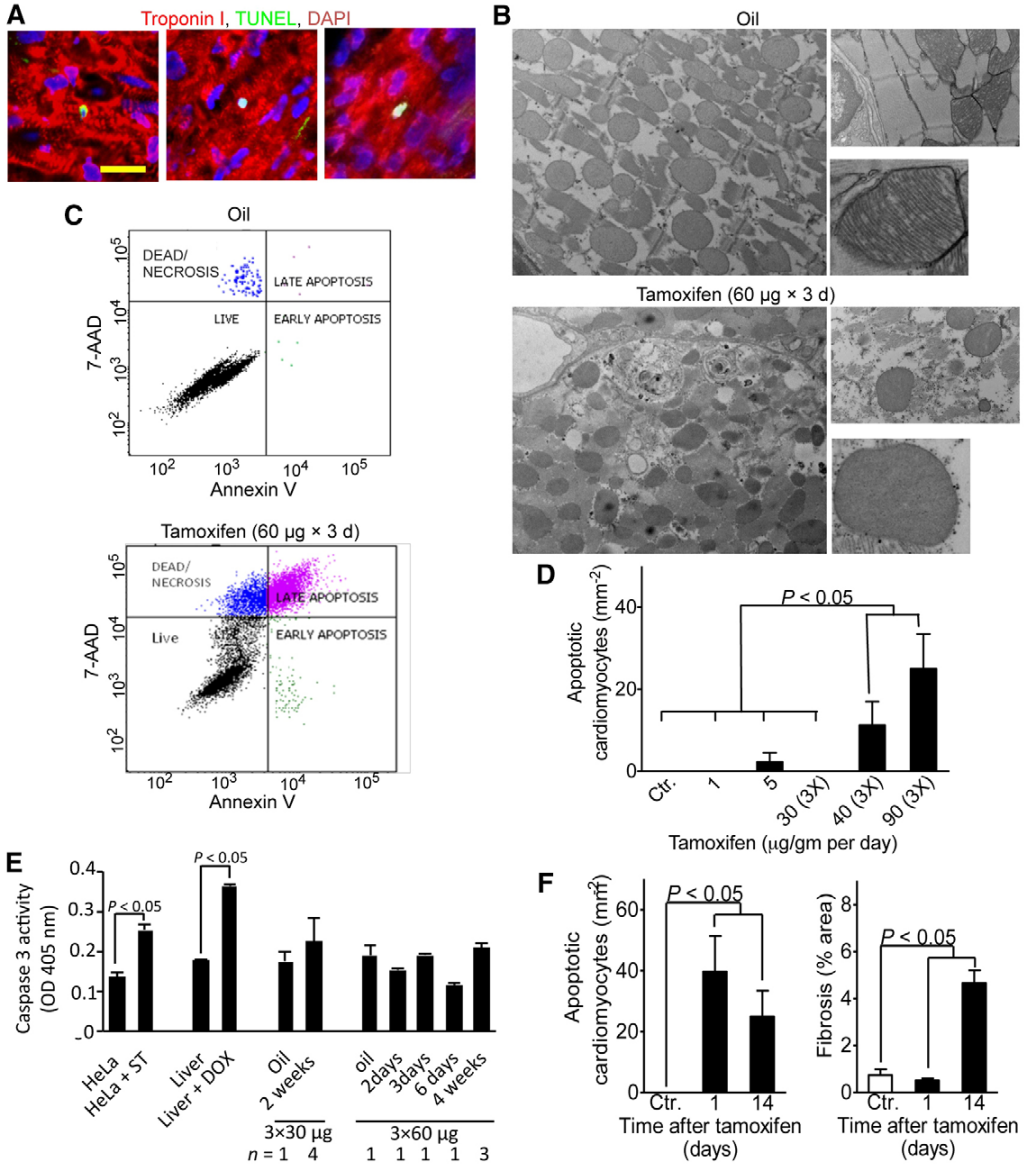
**Tamoxifen injections induce cardiomyocyte apoptosis in *αMHC-MerCreMer* mice.***αMHC-MerCreMer*^+/+^ mice received oil or tamoxifen at the indicated doses (in μg/g body weight) and frequencies on consecutive days. (A) Representative TUNEL stainings after injection of 3×90 μg tamoxifen/g body weight show apoptotic cardiomyocyte nuclei surrounded by organized and disorganized sarcomeres. Scale bar: 25 μm. (B) Electron microscopy showed disorganization of mitochondria, sarcomere disarray, mitochondrial rupture and distorted mitochondrial matrix/cristae after injection of 3×60 μg tamoxifen/g body weight. (C) Flow cytometry of isolated cardiomyocytes stained with annexin V and 7-amino-actinomycin (7-AAD) showed viable cardiomyocytes with a small population of necrotic cardiomyocytes in oil-injected control mice. Cardiomyocytes from tamoxifen-treated mice were viable (annexin-V- and 7-AAD-negative), in early apoptosis (annexin-V-positive and 7-AAD-negative), in late apoptosis (annexin-V- and 7-AAD-positive) or in necrosis (annexin-V-negative and 7-AAD-positive). (D) Quantification at 2 weeks after tamoxifen injection shows increased cardiomyocyte apoptosis at doses above 3×30 μg/g body weight. (E) Quantitative analysis of activated caspase-3 activity following injection of tamoxifen shows no increased caspase activity. Positive controls were HeLa cells treated with staurosporine (ST) and liver from animals injected with doxorubicin (DOX). Negative controls were littermates injected with oil (B–E). (F) Comparing apoptosis and fibrosis between 1 and 14 days after 3×90 μg tamoxifen/g body weight shows that apoptosis precedes widespread fibrosis formation. *n*≥4 animals per group (D,F); number of animals indicated below the *x*-axis (E). Statistical significance tested by ANOVA.

Previous reports have suggested that Cre can induce apoptosis (Forni et al., 2006; Naiche and Papaioannou, 2007; Silver and Livingston, 2001; Buerger et al., 2006). Thus, we quantified apoptotic cardiomyocytes at the same time as fibrosis, 2 weeks after tamoxifen administration. We detected TUNEL-positive nuclei that were completely surrounded by troponin I signal in a striated pattern, suggestive of apoptotic cardiomyocytes that have not yet disassembled their sarcomeres (Fig. 3A). We also observed apoptotic cardiomyocytes with diffuse troponin I staining, indicative of sarcomere disassembly or degradation. We evaluated the involvement of mitochondrial changes in *αMHC-MerCreMer* mice after injection of 3×60 μg tamoxifen/g body weight. Electron microscopy showed disorganization of mitochondria, distorted mitochondrial cristae and mitochondrial rupture, which are characteristic of cardiomyocyte apoptosis (Fig. 3B). We then determined the appearance of phosphatidylserine in the outer leaflet of the sarcolemma, which is indicative of early apoptosis, with the annexin V assay (Fig. 3C). In oil-injected control mice, flow cytometry of isolated cardiomyocytes stained with annexin V and 7-amino-actinomycin (7-AAD; to identify necrotic cardiomyocytes) showed viable cardiomyocytes and a small population of necrotic cardiomyocytes. In contrast, tamoxifen-injected mice had a larger population of cardiomyocytes in early apoptosis (annexin-V-positive and 7-AAD-negative), in late apoptosis (annexin-V- and 7-AAD-positive) or in necrosis (annexin-V-negative and 7-AAD-positive). The preponderance of cardiomyocytes in early and late apoptosis in tamoxifen-treated *αMHC-MerCreMer* mice is indicative of Cre-induced apoptosis.

We determined whether cardiomyocyte apoptosis was dependent on the degree of activation of the *αMHC-MerCreMer* system. Quantification of TUNEL-positive cardiomyocytes showed that single tamoxifen doses of 1 or 5 μg/g body weight in *αMHC-MerCreMer* mice did not increase cardiomyocyte apoptosis over that observed in Cre-negative controls (Fig. 3D). At a tamoxifen dose of 3×30 μg/g body weight, there was no detectable apoptosis (Fig. 3D), although there was a non-significant increase in cardiac fibrosis at this time (Fig. 2D). We observed increased apoptosis in both the 40 and 90 μg tamoxifen/g body weight doses (*P*<0.05 for both groups; Fig. 3D). In conclusion, inducing Cre with tamoxifen doses of 40 μg/g body weight or higher is associated with cardiomyocyte apoptosis.

Activation of caspases, a family of cytoplasmic proteases, is crucial for the induction and execution of apoptosis. To determine whether caspase activation is involved in Cre-induced cardiomyocyte apoptosis, we determined the activity of caspase 3. Although caspase-3 activity was slightly elevated 2 weeks after administration of 3×30 μg tamoxifen/g body weight, this was not statistically significant (Fig. 3E). Caspase-3 activity after administration of 3×60 μg tamoxifen/g body weight was not increased at various time points. These findings suggest that Cre-induced cardiomyocyte apoptosis might not be a caspase-mediated process (Bae et al., 2008).

Does the cardiomyocyte apoptosis seen in our findings precede the onset of myocardial fibrosis, or is it the result of cardiac dysfunction and fibrosis? In order to address this question, we compared the global extent of apoptosis and myocardial fibrosis immediately after completion of tamoxifen administration and 2 weeks later (Fig. 3F). To induce Cre toxicity, we injected 90 μg tamoxifen/g body weight for three consecutive days and examined these mice 1 day and 2 weeks later. Cardiomyocyte apoptosis was increased at 1 day, but fibrosis was not increased. At 2 weeks, however, apoptosis and fibrosis were both increased (Fig. 3F), indicating that Cre induces apoptosis prior to fibrosis formation. This suggests that apoptosis might be an underlying pathogenic mechanism of Cre-induced myocardial fibrosis.

### High levels of Cre activate a DNA damage response in cardiomyocytes

**Fig. 4.**
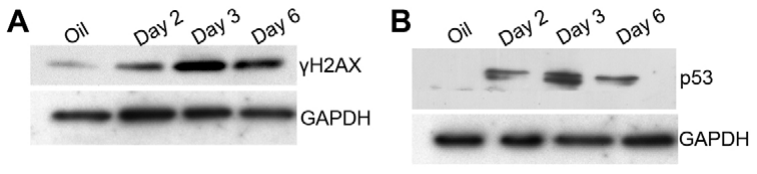
**Tamoxifen injections induce DNA damage response signaling in *αMHC-MerCreMer* mice.***αMHC-MerCreMer*^+/+^ mice received an injection of 60 μg/g body weight of tamoxifen on three consecutive days. Western blot analysis reveals an increase of γH2AX (A) and p53 (B) at various time points. Littermates injected with oil served as a negative control.

We observed cardiomyocyte apoptosis in tamoxifen-injected *αMHC-MerCreMer* mice in the absence of *loxP* alleles, suggesting that the observed effects might be due to DNA breaks at non-*loxP* sites. Double-strand DNA (dsDNA) breaks activate the ataxia telangiectasia mutated (ATM) and ataxia telangiectasia and Rad3-related protein (ATR) pathways, leading to phosphorylation (Ser139) of histone H2X (γ-H2AX). We detected increased γ-H2AX phosphorylation at 2, 3 and 6 days after injecting 60 μg tamoxifen/g body weight (a single injection was given on days 1, 2 and 3), indicating activation of a DNA damage response (Fig. 4A) that coincided with the induction of apoptosis. Stabilization of p53 occurs further downstream of the DNA damage response. We detected acetylation of p53 at 2, 3 and 6 days after injecting 60 μg tamoxifen/g body weight (a single injection was given on days 1, 2 and 3) (Fig. 4B). In summary, high levels of Cre activation in cardiomyocytes induce acute DNA damage response signaling that is independent of site-specific recombination at *loxP* sites.

### Cre-induced cardiomyocyte apoptosis is independent of transgene insertion into the genome

**Fig. 5.**
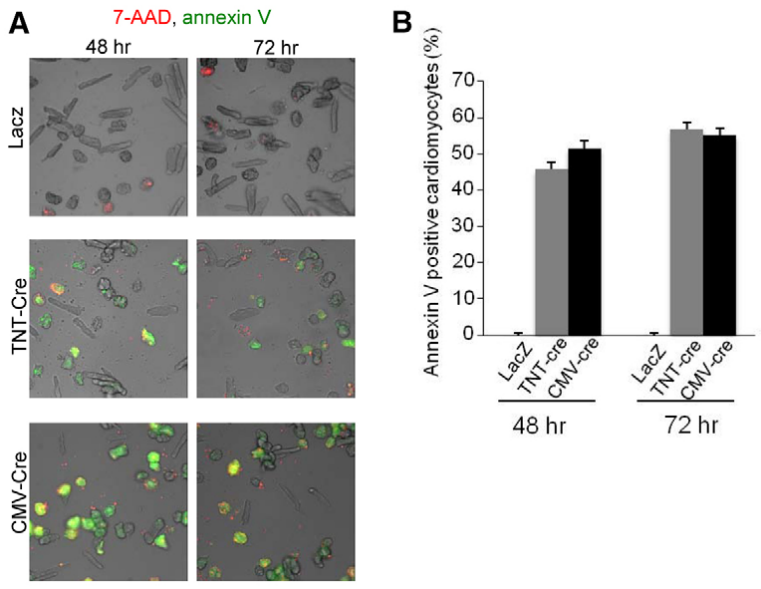
**Transduction with *Cre*-expressing adenoviruses induces cardiomyocyte apoptosis and shows that transgene insertion is not required.** Adult rat ventricular cardiomyocytes were transduced with adenoviruses encoding for *lacZ* or for *Cre* under control of the troponin T promoter (*TNT-Cre*) or CMV promoter (*CMV-Cre*). (A) Whereas necrotic cardiomyocytes (7-AAD-positive) were present in all wells, apoptotic cardiomyocytes (annexin-V-positive) were present in Cre-transduced plates only. (B) Quantification of annexin-V staining showed cardiomyocyte apoptosis associated with *Cre*-expressing adenoviruses.

It is possible that the observed unwanted Cre effects are dependent upon the insertion site of the *αMHC-MerCreMer* construct into the genome. Although the absence of toxicity in the *αMHC-MerCreMer* transgene without tamoxifen strongly argues against this possibility, we directly tested this possibility using transduction of primary adult rat cardiomyocytes with Cre. We used adenoviruses expressing Cre under control of the strong and ubiquitous cytomegalovirus promoter (*CMV-Cre*) and under control of the troponin T promoter (*TNT-Cre*). In control plates transduced with a *lacZ* (encoding β-galactosidase) adenovirus, cardiomyocyte viability was not affected (Fig. 5A). However, *TNTCre* and *CMV-Cre* significantly increased cardiomyocyte apoptosis (Fig. 5A,B). In conclusion, Cre-induced apoptosis does not depend on transgene insertion into the genome, species or promoter used.

### Development of optimal conditions for tamoxifen induction of the *αMHC-MerCreMer* system

**Fig. 6.**
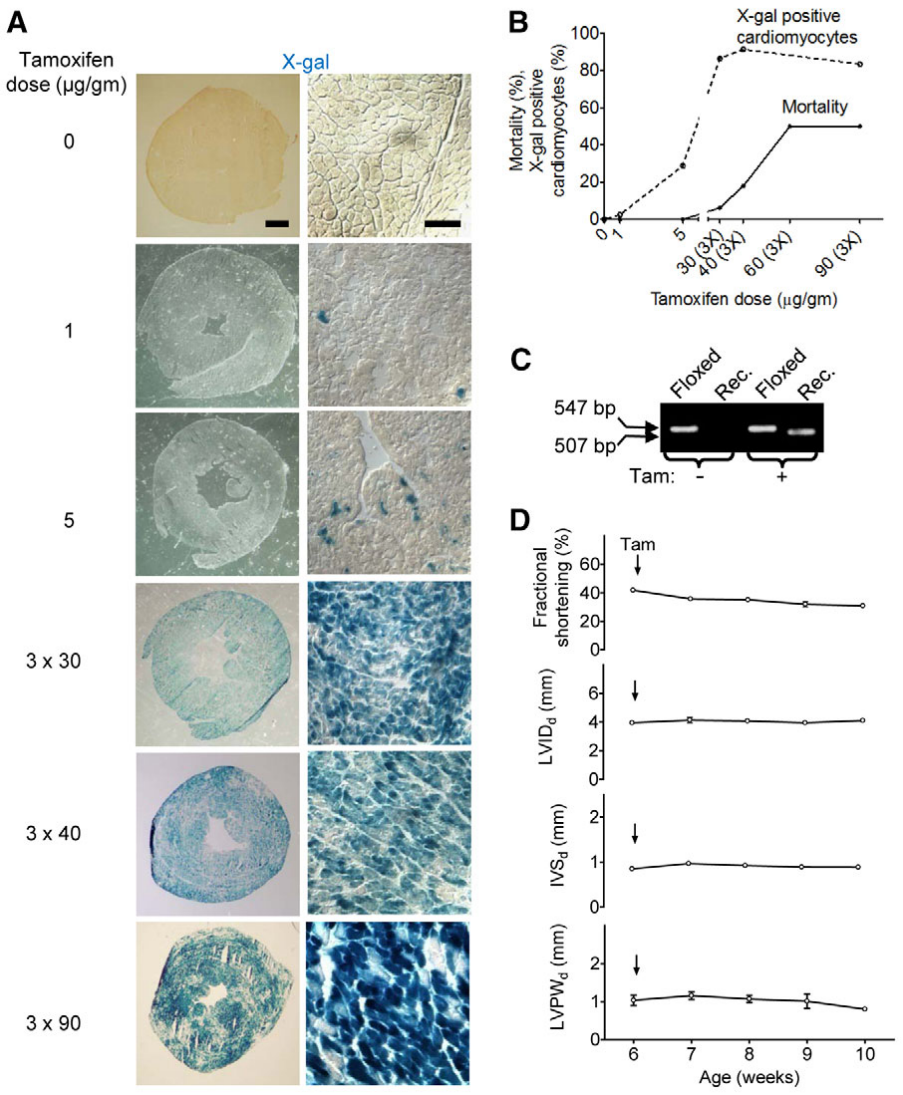
**Site-specific recombination and toxicity occur at different levels of Cre recombinase activity in *αMHC-MerCreMer* mice, revealing a ‘therapeutic window’.** Experiments were performed in *αMHC-MerCreMer*^+/+^; *Rosa26R*^+/–^ mice. (A) Representative examples of micrographs after X-gal staining. Scale bars: 1 mm and 100 μm for left and right panels, respectively. (B) Dosing 3×30 μg tamoxifen/g body weight maximizes site-specific recombination while minimizing cumulative 14-day mortality. (C) The efficiency of creating genomic recombination was determined in *αMHC-MerCreMer*^+/+^; *ErbB4^flox/flox^* mice after injection of 3×30 μg tamoxifen/g body weight. Genomic PCR shows ∼50% recombination. The sizes of the specific PCR products from the floxed allele (547 bp) and from the recombined allele (Rec.; 507 bp) are indicated with arrows. Tam, tamoxifen. (D) Injecting 3×30 μg tamoxifen/g body weight (indicated by vertical arrows) does not induce delayed-onset cardiomyopathy as determined by serial echocardiography for 4 weeks. Differences between baseline at 6 weeks of age and follow-up echocardiographs were not statistically significant (*n*=6 per group, ANOVA).

Our findings lead to the following question: given that Cre toxicity is dose-dependent, can a tamoxifen protocol be developed that minimizes toxicity while maximizing site-specific recombination? To address this problem, we determined recombination efficiency and mortality over a range of tamoxifen doses. To visualize site-specific recombination, we bred *αMHC-MerCreMer*^+/+^; *Rosa26R*^+/+^ mice, in which a Cre-sensitive allele of *lacZ* reports site-specific recombination, which can be revealed by X-gal staining (Soriano, 1999). We administered tamoxifen at different doses and frequencies, and quantified X-gal-positive cardiomyocytes 2 weeks after injecting tamoxifen (Fig. 6A). A single dose of tamoxifen at 15 μg/g body weight yielded 151±59 (s.d., *n*=2 mice) X-gal-positive cardiomyocytes per field of view and a single dose of 30 μg/g body weight yielded 137±57 (s.d., *n*=2 mice) per field of view. Thus, doubling the dose of tamoxifen given as single injection did not increase the recombination efficiency. Therefore, we increased the number of doses in order to increase the recombination efficiency. We achieved a recombination efficiency of 83.5±2.3% X-gal-positive cardiomyocytes with three doses of tamoxifen of 30 μg/g body weight each (Fig. 6B). Increasing the dose of tamoxifen above 30 μg/g body weight did not result in higher recombination rates. Cumulative mortality at 14 days after tamoxifen injection was 0 for 1 and 5 μg/g body weight single doses, low for three doses of 30 μg/g body weight, and significant for three doses of either 40, 60 or 90 μg/g body weight (Fig. 6B).

Different genes engineered with *loxP* sites can show differential sensitivity to Cre. To evaluate this possibility, we determined whether 3×30 μg tamoxifen/g body weight shows sufficient recombination on another allele. Using a strain with a floxed exon 2 of the *ErbB4* gene (Golub et al., 2004), we determined that, at the level of genomic DNA, there was at least 50% recombination (Fig. 6C). In summary, these results indicate that injecting 3×30 μg tamoxifen/g body weight achieves maximal recombination efficiency with minimal mortality. It is important to note that this dose and frequency did not affect cardiac function or structure (Fig. 1C,D).

Because we observed a non-significant increase in myocardial fibrosis after 3×30 μg tamoxifen/g body weight, we determined whether these mice showed a delayed change in cardiac function. We tracked function and structure by echocardiography for 4 weeks (Fig. 6D). We did not observe a significant change of FS, LVID, IVS and LV posterior wall (LVPW) thickness, indicating that inducing Cre activity with 3×30 μg tamoxifen/g body weight did not cause delayed onset of cardiomyopathy.

## DISCUSSION

**Fig. 7.**
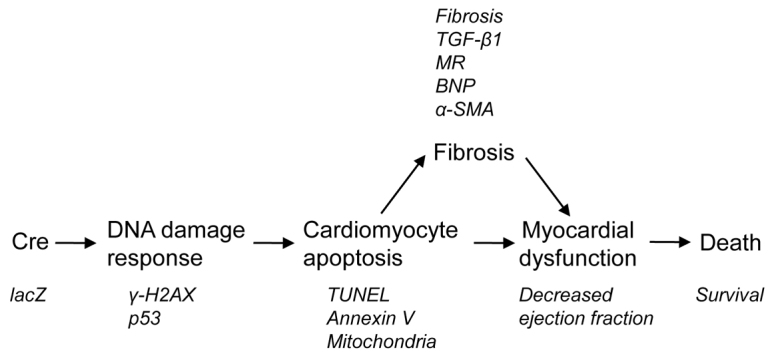
**Tamoxifen injections in *αMHC-MerCreMer* mice induce a *loxP*-independent DNA damage response, leading to myocardial dysfunction and death.** The proposed sequential model of molecular, cellular and tissue mechanisms is provided in the flow diagram. The measured parameters indicating involvement of the proposed mechanisms are indicated in smaller italicized font.

Our study demonstrates that activating nuclear Cre recombinase activity with tamoxifen might cause acute toxicity in cardiomyocytes, and this effect is independent of *loxP* sites. We provide four lines of mechanistic evidence: first, high levels of Cre induction are associated with lethal heart failure in *αMHC-MerCreMer* transgenic mice. Second, Cre induction with three doses of >30 μg tamoxifen/g body weight is associated with myocardial fibrosis. Third, inducing Cre with three doses of >30 μg tamoxifen/g body weight is associated with cardiomyocyte apoptosis. Fourth, inducing Cre with three doses of >30 μg tamoxifen/g body weight is associated with activation of a DNA damage response. Our results also indicate that the DNA damage response and apoptosis are early cellular reactions to Cre recombinase activity in cardiomyocytes, suggesting that they might be pathogenic mechanisms leading to myocardial fibrosis. This mechanistic insight leads us to propose a pathogenic model of *loxP*-independent Cre effects in cardiomyocytes (Fig. 7).

Cellular toxicity induced by non-mammalian transgenes in cardiomyocytes has been described for green fluorescent protein (Huang et al., 2000) and Cre (Buerger et al., 2006). Our present results and previous reports (Koitabashi et al., 2009; Buerger et al., 2006; Hall et al., 2011) highlight the sensitivity of cardiomyocytes to high levels of Cre activity. Because, in the absence of tamoxifen, *αMHC-MerCreMer*^+/+^ mice had normal cardiac function, one can conclude that the presence of Cre in the cytoplasm is not toxic. This makes overload with Cre protein less likely to be responsible for the observed phenotype. These results are consistent with prior reports of reduced cardiac function due to induction of the *αMHC-MerCreMer* system (Koitabashi et al., 2009; Hall et al., 2011). Our study provides additional insight into the cellular and tissue mechanisms.

Cre has been shown to be toxic in proliferating undifferentiated cells (Takebayashi et al., 2008; Naiche and Papaioannou, 2007; Forni et al., 2006). Our study and the report by Lexow et al. (Lexow et al., 2013) are further additions to a list of differentiated cells that are susceptible to Cre toxicity, including insulin-producing β-cells (Lee et al., 2006). How does nuclear Cre activity induce apoptosis in non-dividing cells? Our results show that Cre activates a DNA damage response in the absence of *loxP* sites, suggesting that Cre might induce illegitimate DNA breaks, i.e. at sites other than *loxP* (Semprini et al., 2007; Thyagarajan et al., 2000). In fact, chaotic chromatin recombination has been shown to occur in other differentiated cells, such as spermatids (Schmidt et al., 2000), in which Cre toxicity is evident as chromosomal rearrangements during metaphase. In conclusion, chromosome segregation during cell division might not be required for illegitimate Cre activity, as was suggested by studies in mouse embryonic fibroblasts (Loonstra et al., 2001). An important question for the cardiovascular research community is whether other myocardial cells, including stem and progenitor cells and cardiac fibroblasts in the developing and adult heart, are susceptible to Cre toxicity.

The finding that Cre-induced cardiomyocyte apoptosis did not involve caspase 3 activation was unexpected. However, there is evidence suggesting that caspase-independent pathways play a role in cardiomyocyte death (Bahi et al., 2006; Bae et al., 2008; Kubasiak et al., 2002). The potential importance of caspase-independent pathways in the heart is highlighted by the fact that cardiomyocytes contain high levels of endogenous caspase inhibitors, which render them relatively resistant to caspase-dependent apoptosis (Bae et al., 2008; Bahi et al., 2006; Kubasiak et al., 2002). In addition, caspase inhibition might not be able to completely inhibit apoptosis in cardiomyocytes, thus supporting the notion that Cre-mediated cardiomyocyte apoptosis may be caspase-independent (Okamura et al., 2000; Choudhury et al., 2010).

It is not surprising that the observed *loxP*-site-independent Cre activity was associated with activation of a DNA damage and p53 response. This is in agreement with Cre-dependent induction of apoptosis in *loxP*-containing cells in a p53-dependent mechanism (Zhu et al., 2012).

Recent reports of cardiac Cre toxicity using the *αMHC-MerCreMer* strain (Hougen et al., 2010; Hall et al., 2011; Koitabashi et al., 2009) highlight the importance of experimental design and characterization of the chosen conditions. Cell culture studies performed on embryonic stem cells have shown that Cre cell lines have to be calibrated prior to use in order to understand the differences in toxicity and recombination efficiency that are inherent to each Cre system (Hameyer et al., 2007). This is of particular importance if the gene under investigation has a known or possible role in DNA repair or cell survival. To be able to parse out a potential confounding effect of Cre toxicity, some groups have recommended delaying the analysis of the role of inactivating a gene for at least 1 month after tamoxifen-induced Cre activity (Higashi et al., 2009). However, this limits the possibility of studying the acute response to induced gene deletion. Self-excising Cre constructs might avoid chronic toxicity, but not completely eliminate acute toxicity (Silver and Livingston, 2001).

An important question that arises each time when a modification of the mouse genome shows a phenotype is whether the insertion site contributes to the observed phenotype. The *αMHC-MerCreMer* system is activated by tamoxifen, which provides an opportunity to control the function of the transgene. Our experiments using oil injection as control and using adenoviral transduction with Cre show that Cre toxicity is independent of transgene insertion.

Many of the concerns regarding non-specific outcomes of Cre activity can be addressed by using the appropriate Cre-containing controls and carefully evaluating the Cre strain before use. As our study demonstrates, increasing Cre activity with tamoxifen at doses higher than 30 μg/g body weight did not increase site-specific recombination in *Rosa26-lacZ* mice above 80%, suggesting a ceiling effect of site-specific recombination. Thus, the amount of catalytically active Cre might not be the limiting factor for the site-specific recombination efficiency seen with the *αMHC-MerCreMer* system in combination with the *Rosa26-lacZ* (Bersell et al., 2009) and Z/EG (Hsieh et al., 2007) strains. Our current report shows that injecting tamoxifen at a dose of 30 μg/g body weight is a rational choice, leading to optimized site-specific recombination and reduced toxicity. Moreover, we did not observe decreased myocardial function after 3×30 μg tamoxifen/g body weight.

In experiments using inducible Cre lines to activate a permanent genetic label for cellular fate mapping (Kretzschmar and Watt, 2012), Cre toxicity might affect the longevity, proliferation and differentiation of cells, which in turn could influence the results. The appropriate control would be a Cre-independent experiment, which is often not feasible because the genetic label would not be created.

Using different promoters to drive Cre expression, or even using the same transgene construct inserted in multiple copy numbers at different sites, might lead to different expression levels (Buerger et al., 2006), which will influence the likelihood of toxic Cre effects. Additional known and unknown parameters, including genetic factors (such as strain), environmental factors and nutrition, might influence the multiple aspects of Cre toxicity summarized in Fig. 7. Thus, dosing and timing of tamoxifen administration that we recommend here should be carefully re-evaluated under the specific conditions present in other laboratories.

## MATERIALS AND METHODS

### Mouse strains

The Boston Children’s Hospital Institutional Animal Care and Use Committee approved all of the animal experiments. The *αMHC-MerCreMer* mice were obtained from the Jackson Laboratories and originally generated by Dr Jeffrey Molkentin (Sohal et al., 2001). The *Rosa26-lacZ* mice were obtained from Jackson Laboratories and originally generated by Dr Phillippe Soriano (Soriano, 1999). The *ErbB4^loxP/loxP^* strain was obtained from the Mutant Mouse Repository at UC Davis and originally generated by Dr Kent Lloyd (Golub et al., 2004). Genotyping was performed as previously described (Bersell et al., 2009). All mice were crossed to C57/BL6 mice purchased from Taconic Laboratories.

### Echocardiography

Sedation was induced in an environmental chamber with 3% isofluorane and maintained with 1% isofluorane delivered via a nose cone mask. Echocardiography data was obtained using Vivid*i* with a 12.5 MHz probe (Fig. 1C,E, Fig. 5D) and VisualSonics device (Vevo 2100) with a 40 MHz probe (Fig. 1G).

### RT-PCR

Myocardium was homogenized in Trizol. RNA extraction and DNase treatment was performed with the RNeasy Micro Kit (Qiagen) according to the manufacturer’s instructions. cDNA was synthesized from 200 ng total RNA using SuperScript III and random hexamer primers (Invitrogen) according to the protocol provided by the manufacturer. PCR was performed using Eppendorf Realplex^4^ Mastercycler (35 cycles) and SYBR^®^ Green master mix (Invitrogen). All experiments were performed in duplicate. Fold-change expression of target genes versus controls was calculated after normalization to *GAPDH* housekeeping gene with the Pfaffl method (Pfaffl, 2001). The following specific primers were used for cDNA amplification: MR Forward 5′-AAGGCCTAGATATGGAAAGGCGCT-3′; MR Reverse 5′-TGCTGCTCCCTTGAGTACTGTTGT-3′; BNP Forward 5′-CAGCTCTTGAAGGACCAAGG-3′; BNP Reverse 5′-AGACCCAGGCAGAGTCAGAA-3′; TGF-β1 Forward 5′-GTGCGGCAGCTGTACATTGACTTT-3′; TGF-β1 Reverse 5′-TGTACTGTGTGTCCAGGCTCCAAA-3′; α-SMA Forward 5′-TTCAAGGTGCACACACACACACAC-3′; α-SMA Reverse 5′-TGCTGCTGCCACTCTAGTGAGAAA-3′; GAPDH Forward 5′-GTGCTGAGTATGTCGTGGAGT-3′; GAPDH Reverse 5′-GATGGCATGGACTGTGGTCAT-3′.

### Flow cytometric analysis of apoptosis

Samples were stained with phycoerythrin (PE)-annexin V and the vital dye 7-AAD. In apoptotic cells, the membrane phospholipid phosphatidylserine is translocated from the inner to the outer leaflet of the plasma membrane, which can be detected by annexin V binding. Because externalization of phosphatidylserine occurs in the early stages of apoptosis, annexin V staining can identify apoptotic cells earlier than assays based on nuclear changes like DNA fragmentation, and precedes the loss of membrane integrity that accompanies the latest stages of cell death resulting from either apoptotic or necrotic processes. Therefore, staining with PE-annexin V is typically used in conjunction with 7-AAD to allow identification of early apoptotic cells. Viable cells with intact membranes are PE-annexin-V- and 7-AAD-negative, cells that are in early apoptosis are PE-annexin-V-positive and 7-AAD-negative, cells that are in late apoptosis are both PE-annexin-V- and 7-AAD-positive, and the dead cells are 7-AAD-positive and PE-annexin-V-negative. Briefly, cardiomyocytes were resuspended in 100 μl of binding buffer (140 mM NaCl, 2.5 mM CaCl_2_, 10 mM HEPES; pH 7.4), and 5 μl of PE-annexin V and 5 μl of 7-AAD (Pharmingen) were added and incubated at room temperature in the dark for 15 minutes. Then, 400 μl of binding buffer was added and samples were subjected to fluorescence-activated cell analysis. Noncardiomyocytes were excluded by gating.

### Caspase activity assay

Caspase-3 activity was measured on myocardial lysate using synthetic caspase substrate AcDEVD-pNa (acetyl-Asp-Glu-Val-Asp p-nitroanilide, Caspase-3 Colorimetric Assay Kit, BioVision). Tissues were collected and incubated in caspase lysis buffer [20 mM HEPES (pH 7.5), 10 mM KCl, 1.5 mM MgCl_2_, 1 mM EDTA, 1 mM EGTA, 1 mM DTT, 0.1 mM PMSF, 10 μg/ml leupeptin, 2 μg/ml aprotinin] for 15 minutes on ice. They were then mechanically disrupted with mortar and pestle in liquid nitrogen. The homogenates were centrifuged at 14,000 ***g*** for 10 minutes. Protein concentration of the supernatant was determined with the BCA-based assay (Pierce). Equal amounts of protein (50 μg) were incubated in 50 μl of caspase reaction buffer [50 mM HEPES (pH 7.4), 75 mM NaCl, 0.1% CHAPS, 2 mM DTT] with caspase substrate (200 μM final concentration) in a 96-well microplate at 37°C for 60 minutes. Release of pNa was measured at a wavelength of 405 nm with a spectrometer and adjusted to the background. Measurements were performed in triplicate.

### Immunoblotting

Hearts were collected in RIPA buffer [150 mM NaCl, 1% NP-40 or 0.1% Triton X-100, 0.5% sodium deoxycholate, 0.1% sodium dodecyl sulphate, 50 mM Tris-HCl pH 8, supplemented with protease inhibitors (Complete Cocktail, Boehringer Mannheim)] and disrupted with a glass tissue homogenizer on ice and centrifuged at 10,000 ***g*** for 10 minutes. The protein concentration of the supernatant was determined using the Bradford method (Bio-Rad). Samples with equal amounts of protein (50 μg) were separated by 10% SDS-PAGE and transferred to an Immobilon-P transfer membrane (Millipore), which was incubated with antibodies against γ-H2AX (1:1000, Millipore) and p53 (1:1000, Abcam) in 5% nonfat dry milk at 4°C over night. Bound antibodies were visualized with a chemiluminescence detection system (NEN Life Science Products). For loading controls, membranes were incubated with stripping buffer (Pierce) at 65°C for 30 minutes and re-probed with an antibody against GAPDH (1:1000, Abcam).

### Effects of adenoviral Cre expression on apoptosis in cultured primary cardiomyocytes

To prepare cultures of adult rat cardiomyocytes, rats were anesthetized with isoflurane and hearts were removed. The aortic arch was attached to a Langendorff apparatus, and retrogradely perfused with calcium-free perfusion buffer containing minimum essential medium (Joklik’s modification) supplemented with 5 mM taurine, 2 mM creatine, 5 mM HEPES and 20 u/l insulin for 5 minutes. The hearts were then perfused with perfusion buffer containing 0.3% collagenase for 45 minutes, minced and dissociated in incubation buffer (perfusion buffer with 0.2% BSA and 0.3 mM CaCl_2_) containing 0.3% collagenase. The supernatants containing dissociated cardiomyocytes were washed twice with incubation buffer and the cardiomyocyte fractions separated and plated on laminin-coated plates (10 μg/ml) at 2×10^5^ cells/cm^2^ with serum-free DMEM supplemented with 5 mM taurine, 5 mM creatine, 2 mM L-carnitine, 25 mM HEPES and 20 u/l insulin. After 1 hour of plating, unattached (damaged or dead) cells were removed by changing the media. The cells were used after 24 hours of plating. Adult rat cardiomyocytes were transduced with recombinant adenoviruses expressing TNT-Cre, Ad-CMV-Cre or control *lacZ* at a multiplicity of infection (MOI) of 2.

To determine the effect of adenoviral Cre expression on apoptosis in cultured primary adult rat cardiomyocytes, we quantified annexin V staining and cardiomyocyte viability using the 7-AAD exclusion assay. 10 μl/ml annexin V-FITC (Pharmingen) was added directly to the media containing 2 mM CaCl_2_ and incubated at 37°C for 15 minutes. The number of viable cardiomyocytes was calculated by subtracting all 7-AAD-positive cardiomyocytes from the total number of cardiomyocytes. The percentage of apoptotic cardiomyocytes was calculated as annexin-V-positive over 7-AAD-negative cardiomyocytes. Approximately 1000 cells per dish were counted (20× magnification).

### Induction and quantification of recombination

Tamoxifen (Sigma) was dissolved in warm sunflower seed oil at a concentration of 40 mg/ml and injected i.p. at the indicated doses and frequencies. To determine the percentage of cardiomyocytes that showed Cre activity, we crossed the *αMHC-MerCreMer* allele into a *Rosa26-lacZ* strain (Soriano, 1999). We induced recombination with tamoxifen and quantified the number of β-galactosidase-positive cardiomyocytes by X-gal staining. We performed X-gal staining on 14-μm cryosections, fixed them in 0.02% glutaraldehyde for 15 minutes, and developed X-gal staining by incubating in 1 mg/ml 5-bromo-4-chloro-3-indolyl-β-D-galactopyranoside for 12–18 hours. We did not detect β-galactosidase activity in the absence of Cre or in the presence of Cre without tamoxifen injection. In conclusion, the inducible deletion strategy was very efficient and highly specific for differentiated cardiomyocytes. To determine recombination of the *ErbB4^loxP^* allele, we isolated genomic DNA and performed PCR as described (Bersell et al., 2009; Jackson-Fisher et al., 2006).

### Quantification of fibrosis and apoptosis

We visualized myocardium and scar by Acid Fuchsin Orange G (AFOG) staining (Polizzotti et al., 2012). We determined myocardial area and fibrosis by point count of pink and blue regions of tissue, respectively, on images taken at 40× magnification. Cardiomyocyte apoptosis was determined using the ApopTag Red *In Situ* apoptosis detection kit (Chemicon). We visualized nuclei with 4′,6′-diamidino-phenylindole (DAPI, Invitrogen). Activated caspase-3 was quantified on myocardial lysate using the Caspase 3/CPP32 Colorimetric Assay Kit (BioVision).

### Statistical analyses

Investigators (K.B., S.C., M.M., S.A. and B.K.) quantified observations independently from one another and in a blinded manner. Numeric data are presented as mean ± s.e.m. We tested statistical significance with the *t*-test and analysis of variance (ANOVA). Mortality was analyzed using Kaplan-Meier (GraphPad). The α-value was set at 0.05.

### Note added in proof

Since submission of this paper, off-target activity of CRISPR/Cas9 in mammalian cells has been documented, highlighting the need for careful evaluation of unintended effects of expressing bacterial nucleases (Fu et al., 2013; Hsu et al., 2013; Pattanayak et al., 2013).

## Supplementary Material

Supplementary Material
