# Supplementary material for: Moderate and high amounts of tamoxifen in αMHC-MerCreMer mice induce a DNA damage response, leading to heart failure and death
Source: Dis Model Mech. 2013 Aug 7;6(6):1459–69. doi: 10.1242/dmm.010447 (PMC3820268; doi:10.1242/dmm.010447)
Supplement: Supplementary Material [file supp_6_6_1459__index.html]

Moderate and high amounts of tamoxifen in α-MHC-MerCreMer mice induce a DNA damage response, leading to heart failure and death — Moderate and high amounts of tamoxifen in αMHC-MerCreMer mice induce a DNA damage response, leading to heart failure and death — Supplementary Material 

# Moderate and high amounts of tamoxifen in *αMHC-MerCreMer* mice induce a DNA damage response, leading to heart failure and death

## DMM010447 Supplementary Material

**Files in this Data Supplement:**

- **Supplementary Material PDF**
