# Supplementary material for: Moderate and high amounts of tamoxifen in αMHC-MerCreMer mice induce a DNA damage response, leading to heart failure and death
Source: Dis Model Mech. 2013 Aug 7;6(6):1459–69. doi: 10.1242/dmm.010447 (PMC3820268; doi:10.1242/dmm.010447)
Supplement: Supplementary Material [file supp_010447_DMM010447.pdf]

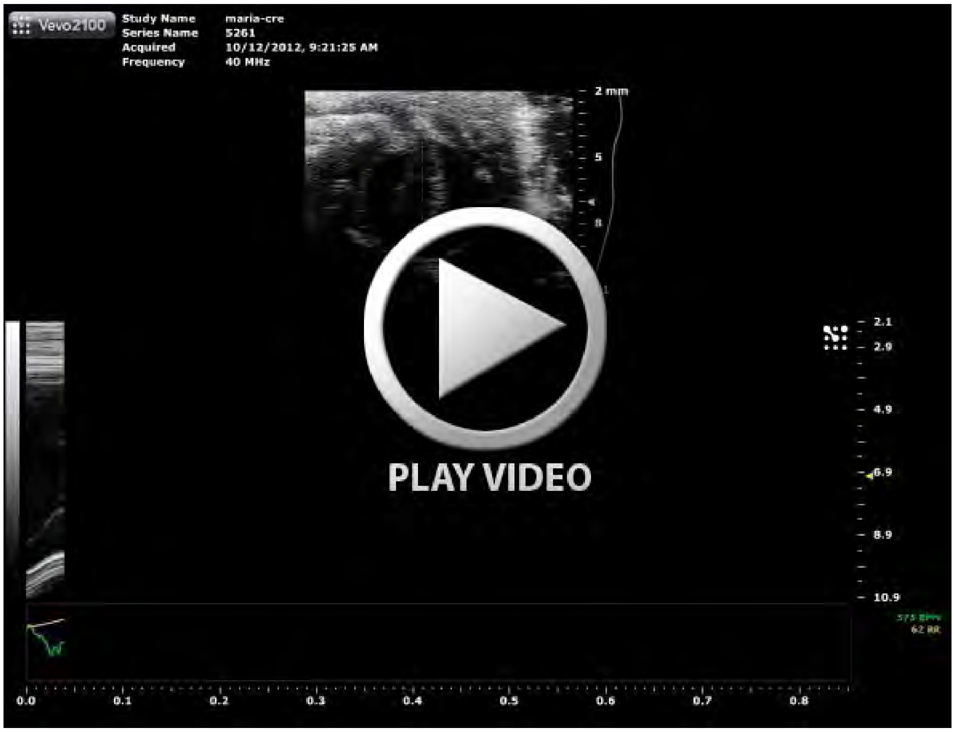

<u>Supplemental Movie S1</u>

Echocardiography was performed 4 weeks after injection of oil into α-MHC-MerCreMer heterozygous mice. This transthoracic echocardiogram in B- and M-mode shows normal cardiac function.

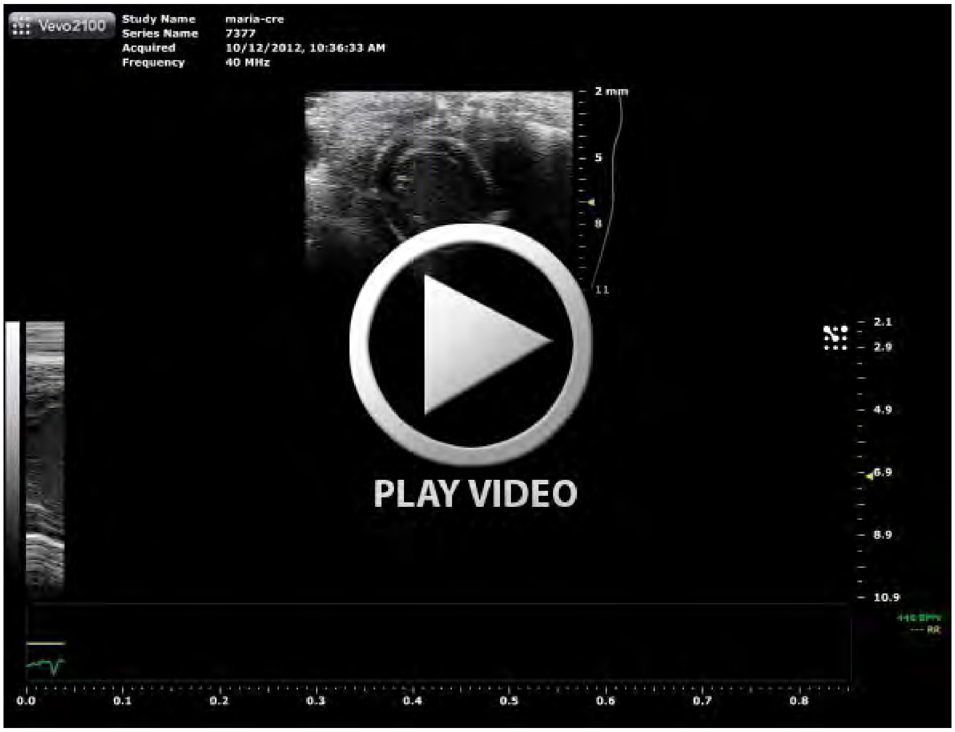

<u>Supplemental Movie S2</u>

Echocardiography was performed 4 weeks after injection of 60 μg/gm of tamoxifen into α-MHC-MerCreMer heterozygous mice. This transthoracic echocardiogram in B- and M-mode shows decrease in cardiac function.
